# Anchoring of FRET Sensors—A Requirement for Spatiotemporal Resolution

**DOI:** 10.3390/s16050703

**Published:** 2016-05-16

**Authors:** Elena V. Ivanova, Ricardo A. Figueroa, Tom Gatsinzi, Einar Hallberg, Kerstin Iverfeldt

**Affiliations:** Department of Neurochemistry, Stockholm University, Svante Arrhenius väg. 16A, SE 10691 Stockholm, Sweden; elena.ivanova@neurochem.su.se (E.V.I.); ricardo@neurochem.su.se (R.A.F.); tom.gatsinzi@gmail.com (T.G.)

**Keywords:** apoptosis, caspase, FRET sensor, live cell imaging, neurodegeneration, signal transduction

## Abstract

FRET biosensors have become a routine tool for investigating mechanisms and components of cell signaling. Strategies for improving them for particular applications are continuously sought. One important aspect to consider when designing FRET probes is the dynamic distribution and propagation of signals within living cells. We have addressed this issue by directly comparing an anchored (taFS) to a non-anchored (naFS) cleavable FRET sensor. We chose a microtubule-associated protein tau as an anchor, as microtubules are abundant throughout the cytosol of cells. We show that tau-anchored FRET sensors are concentrated at the cytoskeleton and enriched in the neurite-like processes of cells, providing high intensity of the total signal. In addition, anchoring limits the diffusion of the sensor, enabling spatiotemporally resolved monitoring of subcellular variations in enzyme activity. Thus, anchoring is an important aspect to consider when designing FRET sensors for deeper understanding of cell signaling.

## 1. Introduction

Signal transduction within living cells is a very complex multilevel process which involves organization of its multiple components into specific microdomains for efficient and tightly controlled signaling [1]. For many signaling molecules, the site and the duration of their activity defines the functional outcome. For instance, transient increases in the concentration of cytoplasmic Ca^2+^ is a key component of synaptic activity and plasticity. On the other hand, prolonged elevation of intracellular Ca^2+^ concentration is a cause of excitotoxicity which leads to neurodegeneration (reviewed by [2]). Similarly, global activation of apoptotic proteases of the caspase family within a cell results in its death, while it has been demonstrated that spatiotemporally limited activation of these enzymes plays important role in differentiation of several types of cells (reviewed by [3]).

Over the past decades, Förster resonance energy transfer (FRET)—based biosensors have proved one of the most valuable and powerful tools for assessing the signal transduction within living cells. For instance, they have been widely used for assaying activity of enzymes, conformational changes in proteins, protein-protein interactions, fluctuations in cytoplasmic ion concentration and pH (reviewed by [4]). Accordingly, FRET probes are continuously being re-designed for more and more advanced applications. One trend is targeting the sensors to different subcellular compartments, which enables monitoring signaling events at the locations of interest (e.g., [5,6,7,8,9,10]). Another direction is improvement of the properties of individual fluorophores and their relative orientation, *i.e.*, FRET efficiency *per se*. However, in the context of the spatiotemporal aspect of signal transduction, anchoring of a sensor is equally important to consider.

We have previously reported detection of caspase-6-like (VEIDase) activity at subcellular levels using a tau-anchored FRET sensor preferentially processed by this caspase [11]. In particular, this sensor allowed for spatial and temporal discrimination between caspase activation within the soma and the neuritic processes of differentiated SH-SY5Y cells. We pursued the aim of investigating initiation and propagation of the enzyme activity with higher precision. To our knowledge, the importance of anchoring *per se* for the performance of FRET sensors has not gained proper attention. Here, we directly compare the properties of two VEIDase sensors which differ only by the presence or absence of the microtubule-associated protein tau. Our results demonstrate that anchoring of FRET sensors is essential for high spatiotemporal resolution.

## 2. Materials and Methods

### 2.1. Design and Construction of FRET Sensors

Plasmid encoding tau-anchored VEIDase sensor (taFS-VEID) based on enhanced cyan fluorescent protein (ECFP)—enhanced yellow fluorescent protein (EYFP) FRET was previously designed and constructed in our laboratory [11]. Plasmid encoding corresponding non-anchored sensor (naFS-VEID) was generated by site-directed substitution of a cytosine to thymidine 12 bases downstream from the start codon of tau-encoding cDNA within taFS-VEID construct. This resulted in an Amber stop codon, TAG, at the 5′-end of tau-encoding sequence. The cloning procedures were performed using QuickChange II Site-Directed Mutagenesis Kit (cat. # 200523, Agilent, Santa Clara, CA, USA) according to the manufacturer’s instructions. The following primers were used: sense, 5′-GGCTGAGCCCCGCTAGGAGTTCGAAGTG-3′, and antisense, 5′-CACTTCGAACT CCTAGCGGGGCTCAGCC-3′.

### 2.2. Cell Culture, Treatment and Transfection

Human neuroblastoma SH-SY5Y and SK-N-AS cells (European Collection of Authenticated Cell Cultures, Salisbury, UK) were cultured in Eagle’s minimal essential medium (MEM) supplemented with 10% fetal bovine serum (FBS), 2 mM glutamine, 1% non-essential amino acids (NEAA), 100 U/mL penicillin and 100 μg/mL streptomycin, from here on referred to as complete medium. The cells were maintained in a humidified 5% CO_2_ atmosphere at 37 °C.

For live cell imaging experiments, the cells were seeded into glass bottom Petri dishes (P35G-0-20-C, MatTek, Ashland, MA, USA), 3–5 × 10^5^ cells/dish, and allowed to attach overnight. The following day the medium was replaced with fresh complete medium and the cells were transfected with plasmids encoding taFS-VEID or naFS-VEID using X-treme Gene HP DNA Transfection Reagent (Roche, Basel, Switzerland) according to the manufacturer’s instructions. SK-N-AS cells were imaged the following day. SH-SY5Y cells were differentiated with retinoic acid (Sigma-Aldrich, St. Louis, MO, USA), diluted to a final concentration of 10 µM in the complete medium, for 3 days following transfection. Prior to the imaging session, the medium was buffered with 10 mM HEPES. When applicable, staurosporine was added to the buffered medium to a final concentration of 1 μM prior to the start of the imaging session. For western blot analysis, SK-N-AS cells were seeded into 6-well plates (Nunclon, Rochester, NY, USA) at a density of 3 × 10^5^ cells/well and allowed to attach overnight. Transfection was performed as described above. Next day, the cells were treated for 3 h with 1 μM staurosporine. All the reagents were purchased from Life Technologies (Carlsbad, CA, USA) if not otherwise indicated.

### 2.3. Live Cell Imaging and Image Analysis

Localization of sensors was determined using an imaging system with a Nipcov spinning disk (CSU-22) on a Zeiss Axiovert 200 using solidstate 488 nm laser illumination (CrystaLaser, Reno, NV, USA) and an Orca-Flash 4.0 sCMOS camera (Hamamatsu Photonics, Shizuoka, Japan). Z-stacks were acquired using a piezo objective actuator (Piezosystem, Jena, Germany) to cover the focal planes of the cells and, for the SH-SY5Y cell protrusions, these focal planes were summed over the *Z*-axis and images linearly adjusted to 0.1% saturation.

Time lapse imaging was performed on a Leica TCS SPI laser scanning confocal microscope (Leica, Heidelberg, Germany) as previously described [11]. ECFP was excited with a 442 nm 10 mW HeCd laser. Emission was collected between 455–505 nm for ECFP and 515–565 nm for EYFP/FRET, using a long pass dichroic mirror 455. Image analysis and calculations were performed using ImageJ version 1.50 g for Windows [12]. For acquiring FRET images, the images from the focal planes from each individual channel were summed over the *Z*-axis, and intensity in the FRET channel was divided by the intensity in the ECFP channel.

Analysis of FRET images was performed by segmenting them into cells and analyzing each cell individually. The pixels of the cells were sorted into percentiles and the ratio value at the 10th percentile and the mean of the 10th–90th percentile was further analyzed, representing the fraction of pixels of the cell retaining the highest FRET and the average FRET, respectively. To correct for the asynchronous apoptosis induction by staurosporine, the data was temporally aligned to the initiation of FRET loss in each cell. The data was temporally interpolated to achieve optimal temporal alignment and subsequently fitted to a mono exponential decay curve using GraphPad Prism version 6.07 for Windows [13].

### 2.4. Western Blot Analysis

Cells were harvested as previously described [11]. 15 μg protein from each sample was separated by electrophoresis on 8%–10% sodium dodecyl sulfate (SDS)-polyacrylamide gels and subsequently transferred to nitrocellulose membranes (GE Healthcare, Little Chalfont, UK) at 350 mA for 2.5 h. The membrane was blocked for 1.5 h in phosphate buffered saline (PBS), containing 5% dry milk (*w*/*v*) and 0.1% Tween, followed by an overnight incubation at 4 °C with rabbit polyclonal anti-GFP antibody (1:2000) (Life Technologies). Finally, the membranes were incubated for 1 h at room temperature with horseradish peroxidase-coupled donkey anti-rabbit (1:5000) (GE Healthcare) and the proteins were visualized by chemiluminescence using SuperSignal™ West Dura Extended Duration Substrate (ThermoFisher Scientific, Waltham, MA, USA).

## 3. Results

### 3.1. Anchored and Non-Anchored FRET Sensors

**Figure 1:**
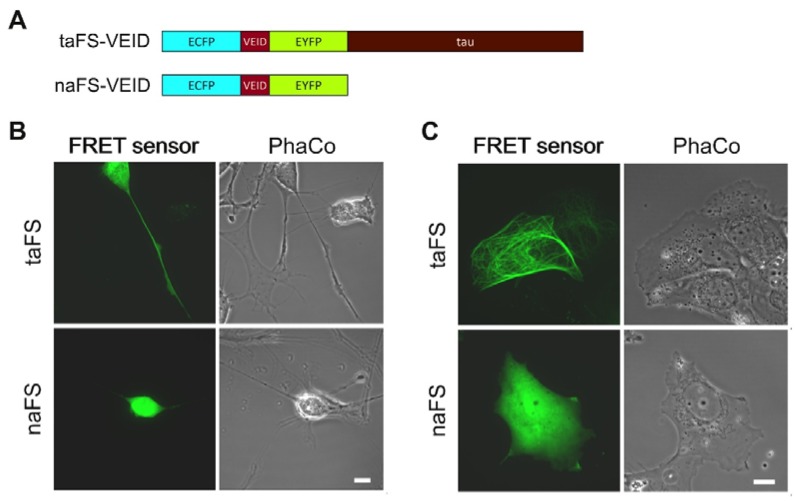
Anchoring enables monitoring of FRET sensors throughout the cell including thin processes. (**A**) Schematic illustration of an anchored (taFS-VEID) and a non-anchored (naFS-VEID) FRET sensors. Both sensors contain a tandem VEID sequence between ECFP and EYFP. naFS-VEID was generated by introducing a stop codon at the 5′-end of tau-encoding sequence within the taFS-VEID cDNA; (**B**) Representative images of differentiated SH-SY5Y cells overexpressing taFS-VEID (upper panel) and naFS-VEID (lower panel); (**C**) Representative images of SK-N-AS cells overexpressing taFS-VEID (upper panel) and naFS-VEID (lower pannel). Note the absence of signal from taFS-VEID, but not naFS-VEID, in the nucleus. The epifluorecence images were linearly adjusted to display 0.1% saturated pixels. Scale bar 10 µm.

In order to evaluate how anchoring of FRET sensors affects their properties, we employed a tau-anchored sensor for monitoring VEIDase activity [11], here designated taFS-VEID (Figure 1A, top). For comparison, we generated a corresponding non-anchored sensor (naFS; Figure 1A, bottom) by introducing a stop codon at the 5′-end of the tau-encoding sequence within the taFS-VEID cDNA.

### 3.2. Anchoring Leads to Enrichment of FRET Sensors at Specific Subcellular Compartments

To compare the intracellular distribution of anchored and soluble FRET sensors, the constructs were transfected into human neuroblastoma SH-SY5Y cells. The cells were subsequently differentiated towards a neuronal phenotype with 10 µM retinoic acid for 3 days, which stimulated outgrowth of neurite-like processes. Analysis was performed by confocal laser scanning microscopy (CLSM). Cells expressing taFS-VEID displayed intense and defined fluorescence throughout the cytoplasm, readily detectable even within thin processes (Figure 1B, top). By contrast, naFS-VEID produced a more dissipated fluorescent signal in the cell bodies, whereas in the processes it could hardly, if at all, be detected (Figure 1B, bottom). Similar observations apply to the neuroblastoma cell line SK-N-AS. These cells form protrusions prior to undergoing staurosporine-induced apoptosis. These protrusions displayed bright fluorescence when overexpressing taFS-VEID, but not naFS-VEID (Figure 1C). Thus, tau-anchoring favors enrichment of FRET sensors in cellular processes.

### 3.3. Tau Anchoring Prevents Nuclear Localization of FRET Sensors

The fluorescent signal from taFS-VEID was lacking in the nuclei of SH-SY5Y cells, as compared to the other compartments, and was clearly absent from the nuclei of SK-N-A-S cells. By contrast, the signal from naFS-VEID was more concentrated in the nuclei of these cells (Figure 1B,C). This provides evidence for the specificity and efficiency of the anchor within taFS-VEID, as tau targets the sensor to microtubules and prohibits its diffusion into the nucleus. By contrast, naFS-VEID, being composed of only ECFP-linker-EYFP, is free to diffuse throughout the cell and enter the nucleus, as has been previously observed for GFP dimers [14]. Thereby, tau anchoring provides specific detection of active caspases in the cytoplasm, where the apoptotic process is known to be initiated [15,16].

### 3.4. Anchoring of FRET Sensors Enables Detection of Active Caspases at Subcellular Level

Next, we proceeded to investigate the properties of anchored and soluble FRET sensors in terms of detecting caspase activation at subcellular level. SK-N-AS cells overexpressing taFS-VEID or naFS-VEID were treated with 1 µM staurosporine and imaged by CLSM with 5 min intervals. FRET was approximated as the ratio between the FRET and the ECFP channels.

**Figure 2:**
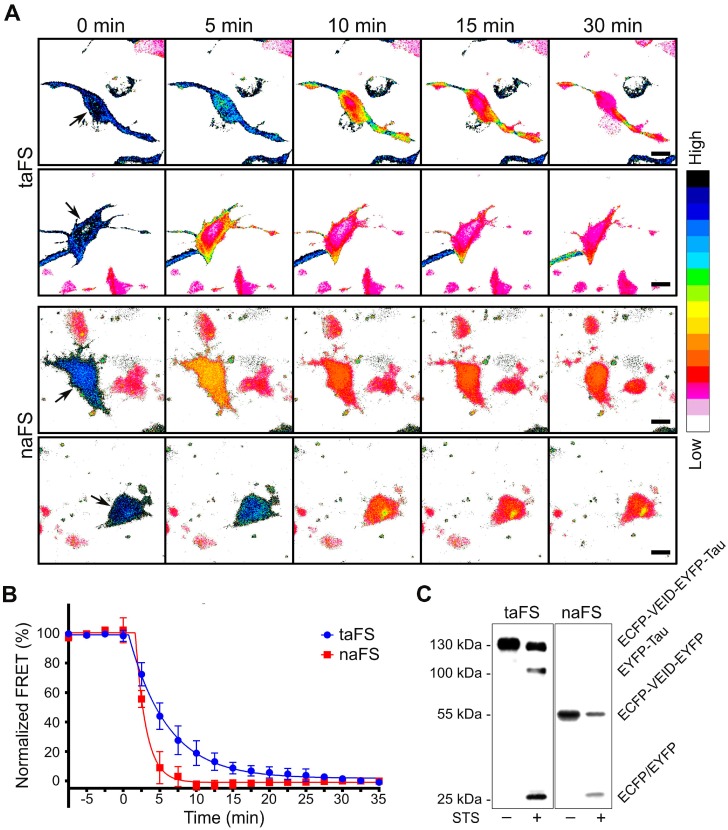
Anchored FRET sensors for detection of active caspases at subcellular level. (**A**) Representative ratiometric (FRET/ECFP) time-lapse images of SK-N-AS cells transfected with taFS-VEID (upper panel) or naFS-VEID (lower panel) and treated with 1 µM staurosporine. Note the local differences in FRET within the cells expressing taFS-VEID. The early decline in FRET in the central parts of the taFS-VEID-expressing cells is likely reflecting liberation of ECFP from the anchorage and its resulting ability to diffuse. Scale bar 10 µm. The video montage of the time lapse images is available as Supplementary materials; (**B**) Average of temporally aligned ratio values of the fraction of pixels from each cell retaining the highest FRET (10th percentile) is plotted over time (*n* = 8 for taFS and *n* = 5 for naFS); (**C**) Apoptotic stimuli induce specific fragmentation of anchored (taFS) and non-anchored (naFS) FRET sensors. Human neuroblastoma SK-N-AS cells overexpressing either of the sensors were treated with 1 µM staurosporine (STS) for 3 h. Total cell lysates were analyzed by western blot with anti-GFP antibodies.

In the cells expressing taFS-VEID, the rates of decline in FRET varied substantially among different regions of the cell, including adjacent areas within the same protrusion (Figure 2A, upper panels). These variations were apparent when analyzing different regions of interest of a cell (see Figure S1A,B in the supplementary materials). By contrast, FRET dropped evenly throughout the cells expressing naFS-VEID and reached a steady state within approximately 10 min from the point of the initial decline (Figure 2A, lower panels and Figure S1C,D in the supplementary materials). This steady state was maintained throughout the cells until their detachment, and no local effects could be detected.

The differences in the local distribution of FRET signal between the anchored and non-anchored sensors were further demonstrated by a dynamic unbiased quantitative analysis of the time-lapse images (Figure 2B and Figure S2). In particular, the rate of change in FRET of the pixels retaining the highest levels was more than twofold higher in the images acquired from the cells expressing naFS-VEID as compared to taFS VEID (Figure 2B), reflecting the presence of distinct subcellular regions with varying degrees of taFS-VEID fragmentation.

Thus, anchoring of FRET sensors enables detection of subtle differences in caspase activation at subcellular level, while the spatiotemporal resolution is lost when using soluble FRET probes.

### 3.5. Specific Proteolysis of VEIDase FRET Sensors

To investigate fragmentation of the FRET sensors, we analyzed total lysates of SK-N-AS cells expressing taFS-VEID or naFS-VEID by western blot using anti-GFP antibodies. Intact taFS-VEID and naFS-VEID sensors migrated as a ~130 kDa and a ~55 kDa band, respectively, matching their theoretical sizes (Figure 2C). Staurosporine-induced apoptosis resulted in fragmentation of taFS-VEID into EYFP-tau (~100 kDa) and ECFP (~25 kDa), while naFS-VEID produced a ~25 kDa fragment representing both ECFP and EYFP. Thus, both taFS-VEID and naFS-VEID are processed specifically within the designed linker region between the fluorophores.

## 4. Discussion

Over the past decades, FRET biosensors have proved to be useful tools for assessing the activity of a variety of enzymes, and have allowed new insights into different signaling pathways. However, FRET probes of conventional type have limited capacity for determining the important spatiotemporal aspect of enzyme activation. Although subcellular targeting of FRET sensors has been widely reported [5,6,7,8,9,10], the impact of localization and anchoring *per se* on their spatiotemporal resolution has not been considered.

Here, we present a case study using tau-anchored (taFS-VEID) and non-anchored (naFS-VEID) caspase (VEIDase) activity reporters. We provide evidence for the presence of an anchor as a necessary condition for sensitive detection of subcellular variations in enzyme activation. We show that anchoring of FRET sensors leads to their enrichment in the targeted location. In our case, tau mediates binding of the VEIDase sensor to the microtubules of neurite-like processes while retaining the availability of the sensor to the whole pool of cytosolic enzymes. Thus, anchoring is expected to enable detection of FRET at the locations of interest with higher signal-to-noise ratio. Furthermore, taFS-VEID, in contrast to naFS-VEID, was undetectable in the nuclei of neuroblastoma cells, implying that possible nuclear VEIDase activity can be neglected when using taFS. Thereby, anchoring of FRET sensors minimizes the impact of the activity of the enzyme in off-target compartments on the readout.

Further, we show that taFS-VEID, but not naFS-VEID, allows for detection of active caspases at subcellular level. In particular, in neuroblastoma cells undergoing apoptosis, taFS-VEID enabled monitoring of local differences in FRET not only within the same cell, but even within the same protrusion. By contrast, no such differences could be observed using naFS-VEID. Accordingly, the rate of change in the cellular regions retaining highest FRET was markedly delayed in case of taFS-VEID. This demonstrates that anchoring enables detection of subcellular variations in the amount of active enzymes. We suggest that restricting the diffusion of FRET sensors by anchoring is advantageous for assessing the propagation of an enzyme activation within a cell, as in this case it is the diffusion of an active enzyme and/or its activating signal that affects the FRET. Conversely, free diffusion of non-anchored FRET sensors makes them uniformly available for an active enzyme throughout the cell, leading to a rapid and global “all-or-nothing” response, which might not reflect the actual pattern of the enzyme activation. Thus, anchoring can be expected to be advantageous not only for cleavable, but also for conformation dependent FRET sensors (e.g., cameleons [17] and Rango [18]), and to visualize cell signaling pathways in more detail.

## 5. Conclusions

In conclusion, our results illustrate the advantage of anchored FRET sensors for assessing activation of enzymes at a subcellular level. The localization and duration of signaling is increasingly recognized as an essential factor determining the functional outcome. Thus, obtaining the spatiotemporal context is important for understanding the mechanisms of cell signaling pathways both in health and in disease, as well as for drug screening. In view of this, it is advisable to consider subcellular anchoring when designing FRET biosensors.

## Supplementary Materials

The following are available online at www.mdpi.com/1424-8220/16/5/703/s1, Figure S1: FRET decay in soma and protrusions. Mean FRET intensity in the indicated regions of interest (ROIs) within SK-N-AS cells overexpressing taFS-VEID (**A**,**B**) or naFS-VEID (**C**,**D**) presented in Figure 2A (see main text) is plotted over time. The data was temporally aligned to the initiation of decline in FRET in each individual cell. For each ROI, the value before the initial decline in FRET was set to 100%, and the lowest FRET value within the analyzed time frame was set to 0%. ROIs 1 represent the soma in all the cells analyzed. Note, in (**A**,**B**) the rate of decline in FRET differs depending on the ROI chosen, whereas there are no such variations between different ROIs in (**C**,**D**), Figure S2: Dynamics of the mean FRET values between the 10th and the 90th percentiles for taFS-VEID and naFS-VEID expressing SK-N-AS cells treated with staurosporine. Videos S1 and S2: Video montages of time lapse image acquisitions from staurosporine-treated SK-N-AS cells expressing anchored (taFS-VEID; Video S1) or non-anchored (Video S2) FRET sensors (see materials and methods). Scale bar 10 µm. Frame interval 5 min.

## Abbreviations

The following abbreviations are used in this manuscript:

CLSM	Confocal laser scanning microscopy
ECFP	Enhanced cyan fluorescent protein
EYFP	Enhanced yellow fluorescent protein
FRET	Förster resonance energy transfer/Fluorescence resonance energy transfer
naFS	Non-anchored FRET sensor
taFS	Tau-anchored FRET sensor
