# Supplementary material for: Anchoring of FRET Sensors—A Requirement for Spatiotemporal Resolution
Source: Sensors (Basel). 2016 May 16;16(5):703. doi: 10.3390/s16050703 (PMC4883394; doi:10.3390/s16050703)
Supplement: Supplementary file 1 [file sensors-16-00703-s001.zip › sensors-16-00703-supplemenatry - update/sensors-16-00703-supplemenatry.pdf]

Supplementary Materials: Anchoring of FRET Sensors—A Requirement for Spatiotemporal Resolution

Elena V. Ivanova, Ricardo A. Figueroa, Tom Gatsinzi, Einar Hallberg and Kerstin Iverfeldt

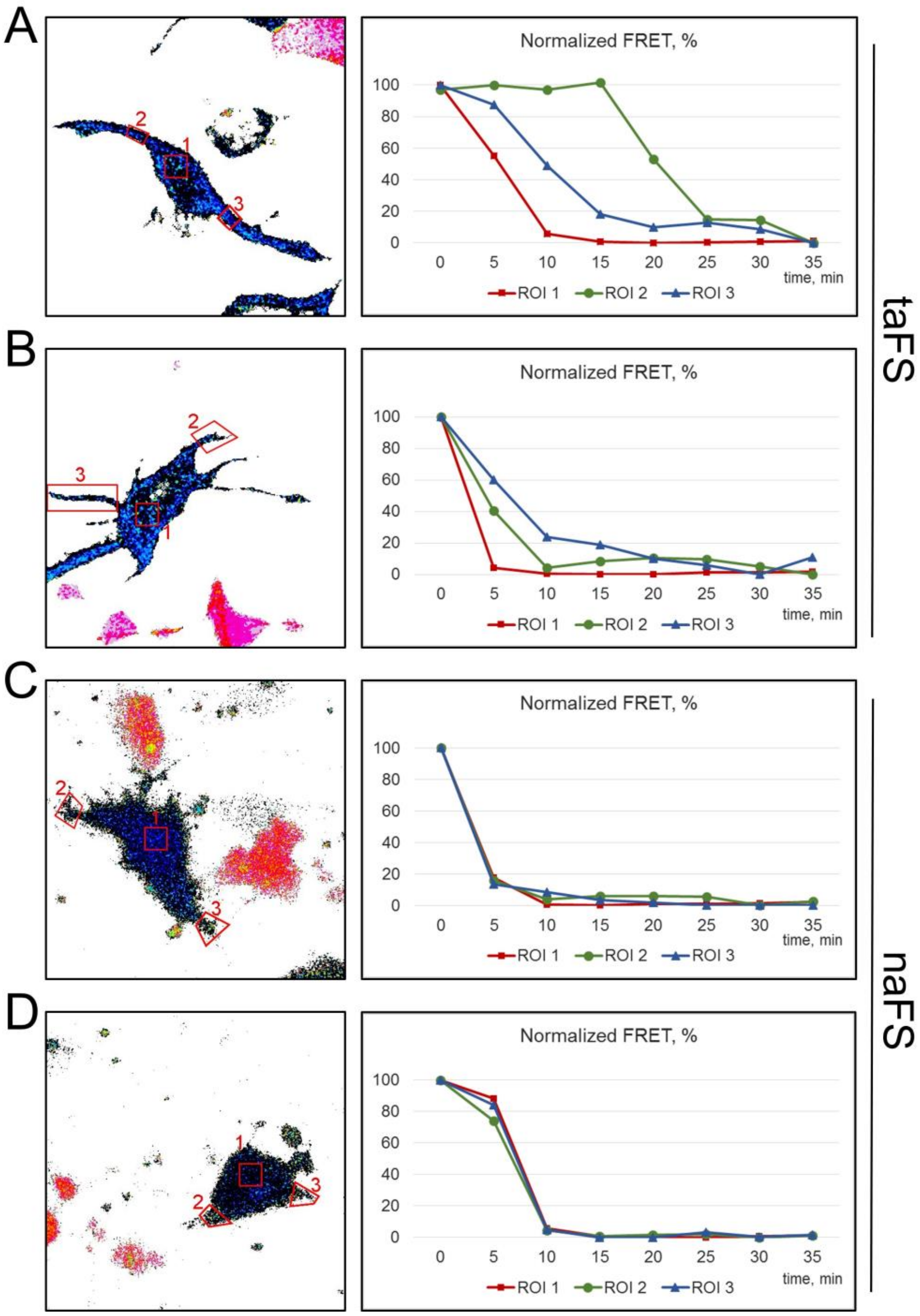

Figure S1. FRET decay in soma and protrusions. Mean FRET intensity in the indicated regions of interest (ROIs) within SK-N-AS cells overexpressing taFS-VEID (**A**,**B**) or naFS-VEID (**C**,**D**) presented in Figure 2A (see main text) is plotted over time. The data was temporally aligned to the initiation of decline in FRET in each individual cell. For each ROI, the value before the initial decline in FRET was set to 100%, and the lowest FRET value within the analyzed time frame was set to 0%. ROIs 1 represent the soma in all the cells analyzed. Note, in (**A**,**B**) the rate of decline in FRET differs depending on the ROI chosen, whereas there are no such variations between different ROIs in (**C**,**D**).

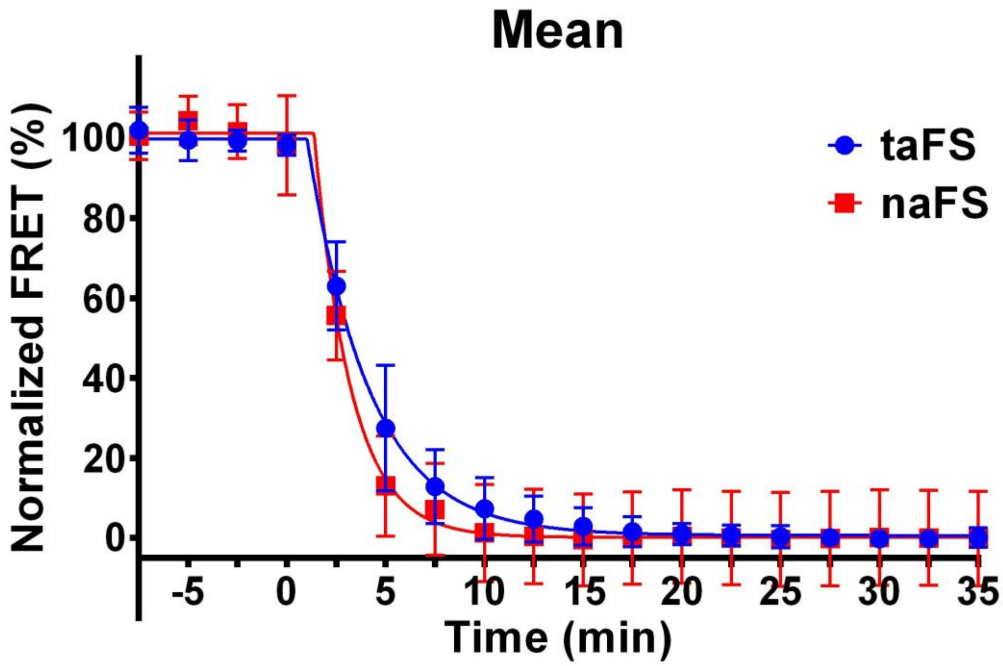

Figure S2. Dynamics of the mean FRET values between the 10th and the 90th percentiles for taFS-VEID and naFS-VEID expressing SK-N-AS cells treated with staurosporine.

Videos S1 and S2. Video montages of time lapse image acquisitions from staurosporine-treated SK-N-AS cells expressing anchored (taFS-VEID; Video S1) or non-anchored (Video S2) FRET sensors (see materials and methods). Scale bar 10 μm. Frame interval 5 min.
